# Associations between household overcrowding and adult mental illness in an ethnically diverse urban population: cross-sectional study using linked primary care and housing records.

**DOI:** 10.23889/ijpds.v9i5.2583

**Published:** 2024-09-18

**Authors:** Marta Wilk, Nicola Firman, Gill Harper, Rich Fry, Lucy Griffiths, Rohini Mathur, Carol Dezateux

**Affiliations:** 1 Queen Mary University of London; 2 Swansea University

## Objectives

We hypothesised that adults living in overcrowded households were more likely to be diagnosed with Serious Mental Illness (SMI) or depression after accounting for individual, household and area-level factors.

## Approach

We linked primary care records of adults currently registered with all general practices in the north-east London region on 21/03/2021 to housing data using pseudonymised unique property reference numbers (pUPRNs), giving a sample of 762,718 adults in 329,948 households. Primary outcomes were a primary care diagnosis of SMI or depression. Overcrowding was defined as <32.5m2 per person. We estimated the adjusted odds (aOR) and 95% confidence intervals (CI) of SMI or depression when living in an overcrowded household, adjusting for individual (age, sex, ethnic group), household (size, composition), and area (Index of Multiple Deprivation) characteristics. We conducted logistic regression models with robust standard errors in RStudio.

## Results

SMI and depression were diagnosed in 6,492 (0.9%) and 45,053 (5.9%) adults respectively; 584,515 (76.6%) adults lived in overcrowded households. In univariable analyses, household overcrowding was inversely related to risk of SMI or depression (aOR[CI]: 0.42 [0.39,0.44]; 0.62 [0.61,0.64]). The direction of effect reversed on adjustment for household size, composition, and area-level deprivation: 1.22 [1.12,1.33]; 1.07 [1.04,1.10] for SMI and depression respectively.

## Conclusions

We found significant associations between SMI and depression and household overcrowding, using a novel method of record linkage to estimate household overcrowding but are not able to establish causality of observed associations in this cross-sectional study.

## Implications

More research is needed to understand how housing conditions affect mental health.

